# Supplementary material for: Using deep learning systems for diagnosing common skin lesions in sexual health
Source: Commun Med (Lond). 2025 Nov 3;5:452. doi: 10.1038/s43856-025-01144-7 (PMC12583615; doi:10.1038/s43856-025-01144-7)

Table S1 Distribution of Images across Different Disease Classes for Model Training, Testing and Evaluation

Data Source	Dataset for Model Training and Validation					Model Evaluation with Prospective Cohort (MSHC June23-Jan24)		
	MSHC (Jan10-May23)	Danderm	Kaggle	Fitz17k	Total	Image Capture App	Digital Camera	Total
**Total (n=16,227)**	**7,892**	**2,993**	**4,230**	**776**	**15,891**	**198**	**138**	**336**
Herpes Simplex Virus	781	62	697	88	1,628	40	19	59
Molluscum Contagiosum	171	12	122	71	376	4	5	9
Monkeypox	141	1	0	0	142	4	0	4
Primary Syphilis (Chancre)	519	2	6	33	560	18	11	29
Secondary Syphilis	153	0	1	31	185	4	0	4
Syphilis Skin Rash	278	10	6	0	294	15	0	15
Genital Warts	1,039	64	657	113	1,873	15	11	26
Gonorrhea/Chlamydia**	233	0	0	7	240	7	32	39
Bacterial Vaginosis	17	3	0	0	20	0	0	0
Penile Papules	52	15	4	0	71	0	3	3
Non-Syphilis Rash	424	202	100	0	726	0	0	0
Balanitis	273	11	5	48	337	17	8	25
Drug Reaction	115	60	137	83	395	4	0	4
Dermatosis	1,193	261	0	0	1,454	15	8	23
Lichenoid Dermatoses	1,186	92	130	14	1,422	21	18	39
Phimosis	28	4	0	0	32	4	2	6
Nevi (Moles)	108	82	0	0	190	0	0	0
Cystic Lesions	18	0	0	7	25	2	0	2
Malignant Lesions	97	118	742	0	957	0	0	0
Vulvar Intraepithelial Neoplasia	167	0	0	0	167	0	0	0
Folliculitis	22	0	63	65	150	2	4	6
Non-STI Lesions*	473	160	0	0	633	11	12	23
Scabies	83	129	0	0	212	9	4	13
Herpes Zoster (Shingles)	121	59	86	0	266	1	0	1
Normal Anatomical Variants	200	3	0	0	203	3	1	4
Dermatitis/Eczema	0	484	0	0	484	0	0	0
Psoriasis	0	170	829	25	1,024	0	0	0
Skin Infections	0	369	75	20	464	1	0	1
Benign Lesions	0	298	0	0	298	0	0	0
Pigmented Malignant Lesions	0	60	570	12	642	0	0	0
Tinea Infections	0	262	0	78	340	0	0	0
Candidiasis	0	0	0	55	55	1	0	1
Chancroid	0	0	0	26	26	0	0	0

** Lesions with no sexually transmitted infection pathogen detected on laboratory testing were included in non-STI lesions.*

*** Images presented with urethral discharges characteristic of gonorrhoea or chlamydia infections in men were included in the Gonorrhoea/Chlamydia category.*

Figure S1 Visualization for 5-Fold Cross-Validation Method for Model Training and Testing

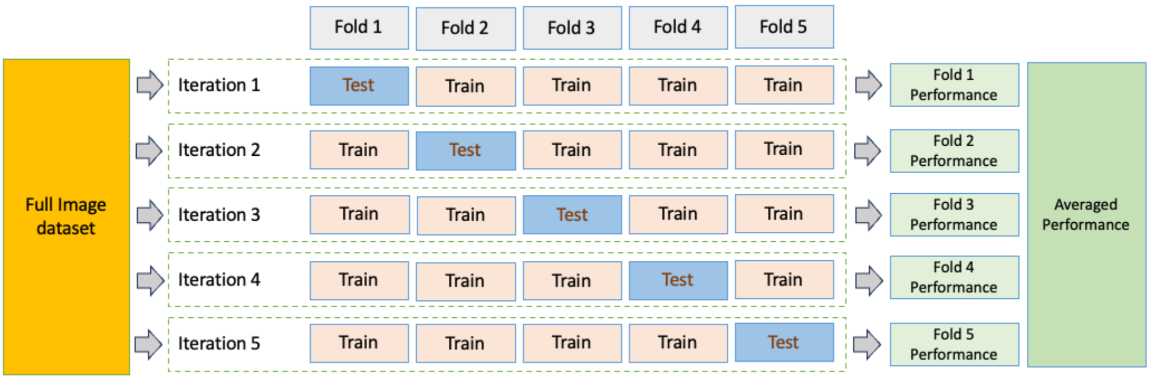

—

Figure S2 Changes in Loss and Accuracy Across Model Training Epoch

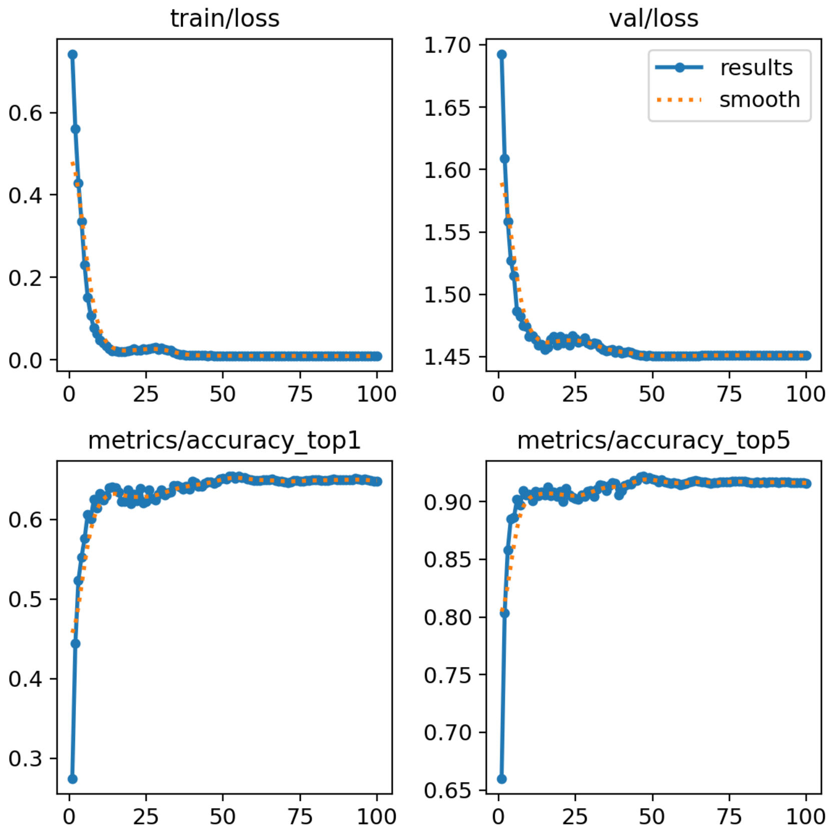

Table S2 Dermatological Conditions and Recommendation for Time to Visit to the Clinician

**Dermatological Conditions**	**Recommendation (Time to visit clinician)**
Herpes Simplex Virus	Within 24 hours
Molluscum Contagiosum	Within next 7 days
Monkeypox	Within 24 hours
Primary Syphilis (Chancre)	Within 24 hours
Secondary Syphilis	Within 24 hours
Syphilis Skin Rash	Within 24 hours
Genital Warts	Within next 7 days
Gonorrhoea/Chlamydia** (presented with discharge)	Within 24 hours
Bacterial Vaginosis	Within next 2-3 days
Penile Papules	Within next 7 days
Non-Syphilis Rash	Within next 2-3 days
Balanitis	Within next 2-3 days
Drug Reaction	Within next 2-3 days
Dermatosis	Within next 2-3 days
Lichenoid Dermatoses	Within next 7 days
Phimosis	Within next 7 days
Nevi (Moles)	Within next 7 days
Cystic Lesions	Within next 2-3 days
Malignant Lesions	Within 24 hours
Vulvar Intraepithelial Neoplasia (VIN)	Within 24 hours
Folliculitis	Within next 2-3 days
Non-STI Lesions* (Pathogen Neg Ulcer)	Within 24 hours
Scabies	Within 24 hours
Herpes Zoster (Shingles)	Within 24 hours
Normal Anatomical Variants	Within next 7 days
Dermatitis/Eczema	Within next 7 days
Psoriasis	Within next 7 days
Skin Infections	Within 24 hours
Benign (Neoplastic) Lesions	Within 24 hours
Pigmented Malignant Lesions	Within 24 hours
Tinea Infections	Within next 2-3 days
Candidiasis	Within next 2-3 days
Chancroid	Within 24 hours

Figure S3A Confusion Matrix of Model Prediction on Training Dataset

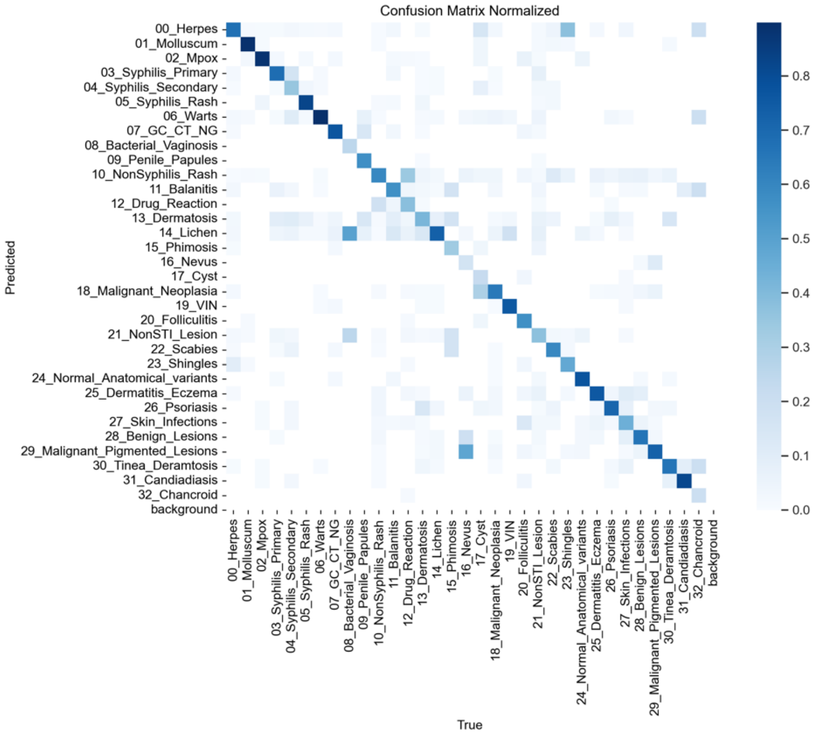

Figure S3B Confusion Matrix of Model Prediction on Testing Dataset

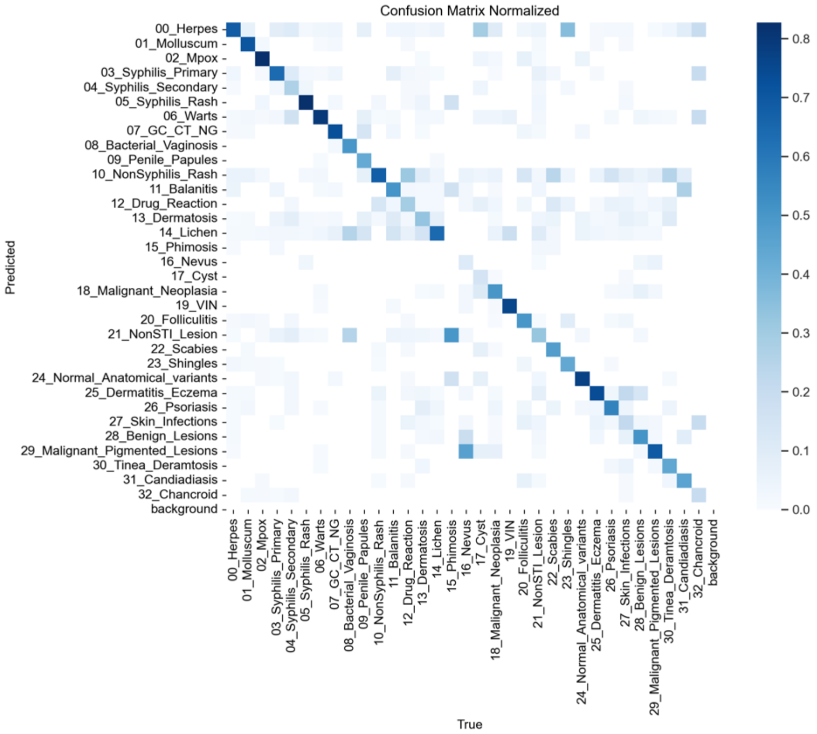

The confusion matrix compares the actual diagnoses along the x-axis to the model's predicted diagnoses along the y-axis.
The diagonal entries (true positive) represent correct predictions while off-diagonal entries represent misclassified cases where the model prediction differed from the actual diagnosis.
The colour scale illustrates the accuracy level. For the diagonal entries, the darker colours indicate to higher accuracy, while lighter colours indicate lower accuracy. For the off-diagonal entries, lighter colours indicate fewer misclassification cases while darker colours indicate more misclassifications.

Figure S4.1 Subgroup Analysis for Model Predictions by Disease Class and Lesion Site

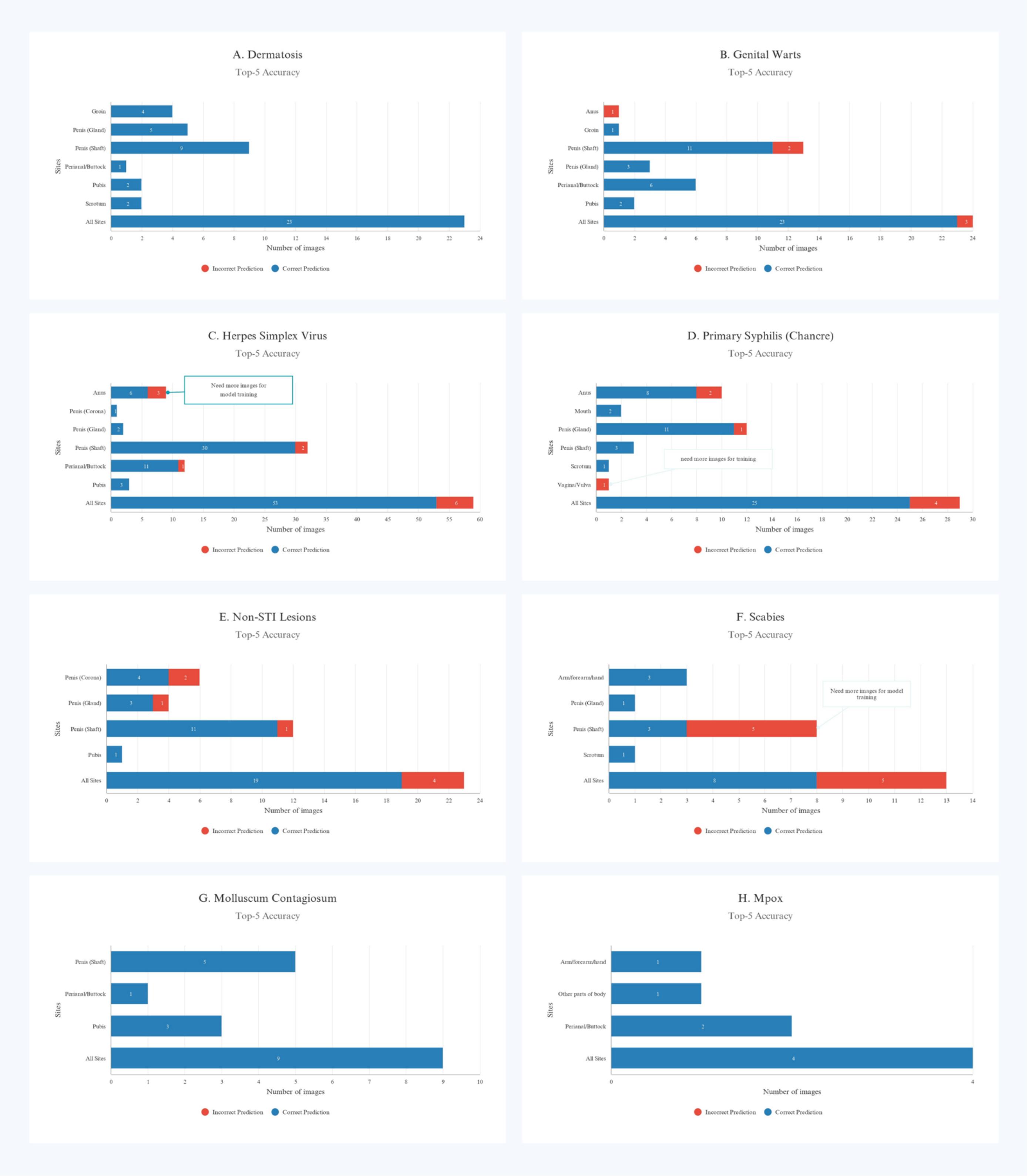

Figure S4.2 Subgroup Analysis for Model Predictions by Disease Class and Lesion Site

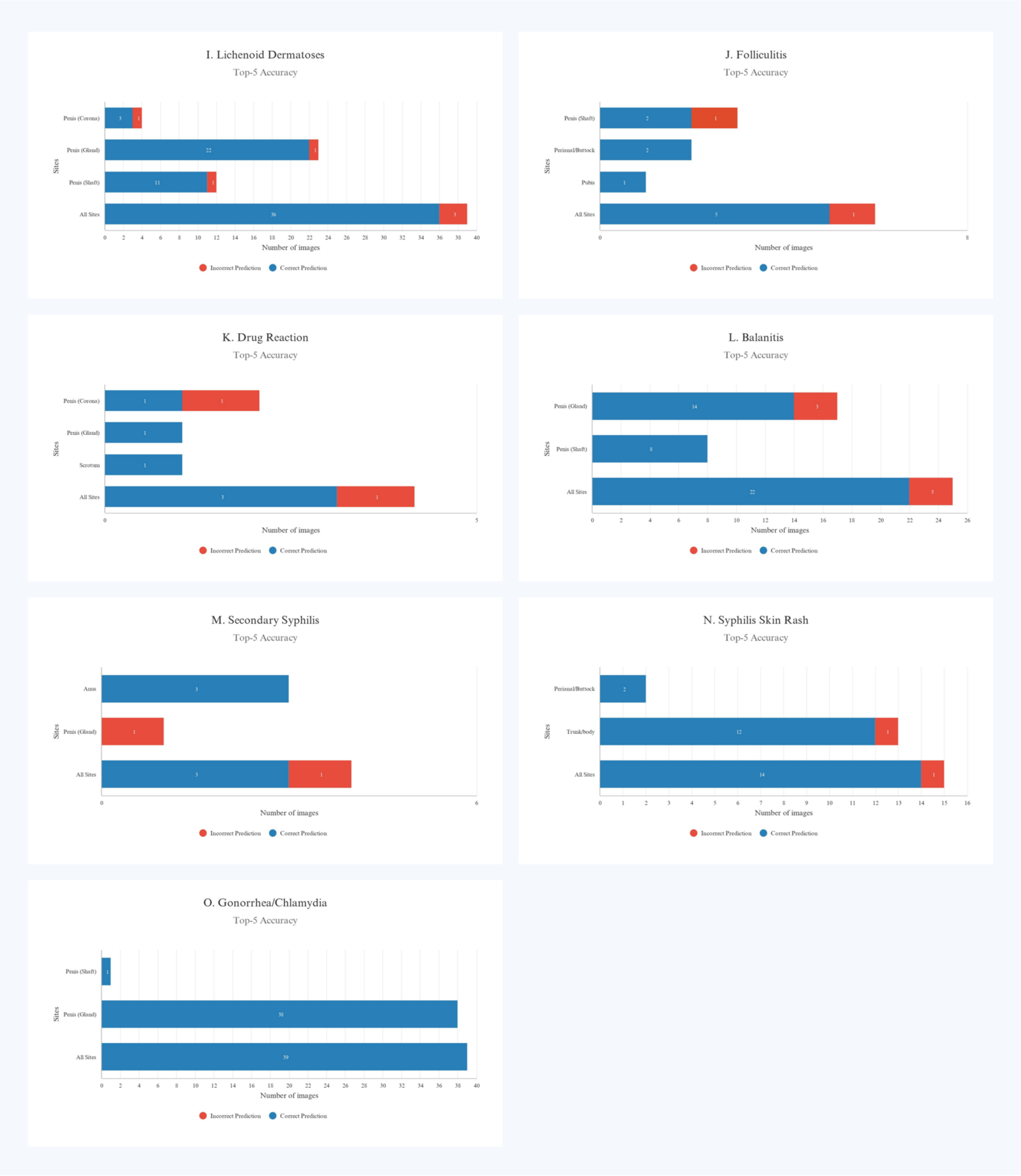

Supplement: Supplementary file 2 — Supplementary Information [file 43856_2025_1144_MOESM2_ESM.pdf]
