# Supplementary material for: Using deep learning systems for diagnosing common skin lesions in sexual health
Source: Commun Med (Lond). 2025 Nov 3;5:452. doi: 10.1038/s43856-025-01144-7 (PMC12583615; doi:10.1038/s43856-025-01144-7)
Supplement: Supplementary file 3 — Description of Additional Supplementary Files [file 43856_2025_1144_MOESM3_ESM.docx]

**Description of Additional Supplementary Files**

File name: Supplementary data 1

Description: Source data for Figure 3

.
