# Supplementary material for: Using deep learning systems for diagnosing common skin lesions in sexual health
Source: Commun Med (Lond). 2025 Nov 3;5:452. doi: 10.1038/s43856-025-01144-7 (PMC12583615; doi:10.1038/s43856-025-01144-7)
Supplement: Supplementary file 5 — Reporting summary [file 43856_2025_1144_MOESM5_ESM.pdf]

nature portfolio

Corresponding author(s): Dr Nyi Nyi Soe, Prof. Lei Zhang

Last updated by author(s): Jul 2, 2025

Reporting Summary

Nature Portfolio wishes to improve the reproducibility of the work that we publish. This form provides structure for consistency and transparency in reporting. For further information on Nature Portfolio policies, see our Editorial Policies and the Editorial Policy Checklist.

Statistics

For all statistical analyses, confirm that the following items are present in the figure legend, table legend, main text, or Methods section.

n/a	Confirmed	
☐	☒	The exact sample size (*n*) for each experimental group/condition, given as a discrete number and unit of measurement
☒	☐	A statement on whether measurements were taken from distinct samples or whether the same sample was measured repeatedly
☒	☐	The statistical test(s) used AND whether they are one- or two-sided<br>*Only common tests should be described solely by name; describe more complex techniques in the Methods section.*
☒	☐	A description of all covariates tested
☒	☐	A description of any assumptions or corrections, such as tests of normality and adjustment for multiple comparisons
☒	☐	A full description of the statistical parameters including central tendency (e.g. means) or other basic estimates (e.g. regression coefficient) AND variation (e.g. standard deviation) or associated estimates of uncertainty (e.g. confidence intervals)
☒	☐	For null hypothesis testing, the test statistic (e.g. *F*, *t*, *r*) with confidence intervals, effect sizes, degrees of freedom and *P* value noted<br>*Give P values as exact values whenever suitable.*
☒	☐	For Bayesian analysis, information on the choice of priors and Markov chain Monte Carlo settings
☒	☐	For hierarchical and complex designs, identification of the appropriate level for tests and full reporting of outcomes
☒	☐	Estimates of effect sizes (e.g. Cohen's *d*, Pearson's *r*), indicating how they were calculated

Our web collection on statistics for biologists contains articles on many of the points above.

Software and code

Policy information about availability of computer code

Data collection	No software was used to collect images from publicly available online repositories. We used the Image Capture app, developed in-house at the Melbourne Sexual Health Centre, to collect clinical images.
Data analysis	We used open-source YOLOv8 image classification package to fine-tune and evaluate our model. Codes are available here "https://docs.ultralytics.com/."

For manuscripts utilizing custom algorithms or software that are central to the research but not yet described in published literature, software must be made available to editors and reviewers. We strongly encourage code deposition in a community repository (e.g. GitHub). See the Nature Portfolio guidelines for submitting code & software for further information.

Data

Policy information about availability of data

All manuscripts must include a data availability statement. This statement should provide the following information, where applicable:

- Accession codes, unique identifiers, or web links for publicly available datasets
- A description of any restrictions on data availability
- For clinical datasets or third party data, please ensure that the statement adheres to our policy

The images of the datasets are available from the following databases. The Danderm dataset is available from https://danderm-pdv.is.kkh.dk/atlas/index.html. The

Kaggle skin lesion dataset can be assessed at https://www.kaggle.com/datasets/shubhamgoel27/dermnet. The Fitz17k is available on GitHub (github.com/mattgroh/fitzpatrick17k). Part of the MSHC dataset is available at https://stiatlas.org/.

Human research participants

Policy information about studies involving human research participants and Sex and Gender in Research.

Reporting on sex and gender	NA
Population characteristics	NA
Recruitment	This study used only de-identified images from publicly available online repositories and from Melbourne Sexual Health Centre.
Ethics oversight	Ethical review was approved by the Alfred Hospital Ethics Committee (Project Number: 524/21 and 683/22). The study was conducted in accordance with the ethical regulations and guidelines.

Note that full information on the approval of the study protocol must also be provided in the manuscript.

Field-specific reporting

Please select the one below that is the best fit for your research. If you are not sure, read the appropriate sections before making your selection.

☒ Life sciences ☐ Behavioural & social sciences ☐ Ecological, evolutionary & environmental sciences

For a reference copy of the document with all sections, see nature.com/documents/nr-reporting-summary-flat.pdf

Life sciences study design

All studies must disclose on these points even when the disclosure is negative.

Sample size	Sample size for this study was not determined using formal statistical methods. In image classification model training, having a larger sample size is advantageous. Therefore, we included all eligible clinical image data to enhance the robustness of our model.
Data exclusions	Any images that did not achieve a diagnostic consensus among the clinicians were excluded from our image dataset. We excluded duplicated and low-resolution images to maintain the quality and uniqueness of the dataset.
Replication	We adhered to the reporting standards for artificial intelligence in health care, outlined in the MINimum Information for Medical AI Reporting (MINIMAR) recommendations.
Randomization	We used a 5-fold cross-validation method to minimize potential bias. This method randomly split the dataset into training (80%) and testing (20%) datasets. We repeated this process five times, each time shuffling the dataset to ensure varied images in each fold.
Blinding	We used the retrospectively collected image data and blinding is not applicable in our study.

Reporting for specific materials, systems and methods

We require information from authors about some types of materials, experimental systems and methods used in many studies. Here, indicate whether each material, system or method listed is relevant to your study. If you are not sure if a list item applies to your research, read the appropriate section before selecting a response.

Materials & experimental systems

n/a	Involved in the study	
☒	☐	Antibodies
☒	☐	Eukaryotic cell lines
☒	☐	Palaeontology and archaeology
☒	☐	Animals and other organisms
☒	☐	Clinical data
☒	☐	Dual use research of concern

Methods

n/a	Involved in the study	
☒	☐	ChIP-seq
☒	☐	Flow cytometry
☒	☐	MRI-based neuroimaging
